# Soil carbon and plant richness relationships differ among grassland types, disturbance history and plant functional groups

**DOI:** 10.1007/s00442-021-04992-x

**Published:** 2021-07-25

**Authors:** B. L. Anacker, T. R. Seastedt, T. M. Halward, A. L. Lezberg

**Affiliations:** 1 City of Boulder Open Space and Mountain Parks, Boulder, CO USA; 2 Institute of Arctic and Alpine Research, University of Colorado, Boulder, CO USA

**Keywords:** Soil carbon, Plant species richness, Grasslands, Functional group, Prairie dogs

## Abstract

Understanding the relationship of soil carbon storage and species diversity in grasslands can provide insights into managing these ecosystems. We studied relationships among soil C and plant species richness within ~ 9700 ha of grasslands in Colorado, US. Using 141 grassland transects, we tested how soil C was related to plant species richness, grassland type, soil texture, and prairie dog presence. Soil C was significantly, positively related to plant species richness, while native perennial graminoid species richness exhibited an even stronger positive relationship. However, the relationship of soil C and plant richness was not found in all three grassland types studied, but instead was unique to the most common grassland type, mixed grass prairie, and absent from both xeric tallgrass and mesic tallgrass prairie. The presence of a single indicator species, *Andropogon gerardii*, showed a significant, positive relationship with soil carbon. Our best possible model explained 45% of the variance in soil C using species richness, grassland type, and their interaction. Surprisingly, soil C was negatively related to soil clay, suggesting that surface clays amplify evaporation and water runoff rather than protecting soil organic matter from decomposition. Soil C was negatively related to prairie dog presence, suggesting that prairie dogs do not enhance soil carbon sequestration; in fact, prairie dog occupied sites had significantly lower soil C, likely related to loss of topsoil from prairie dog colonies. Our results suggest that management for species richness provides the co-benefit of soil C storage, and high clay and prairie dog disturbance compromises both.

**Supplementary Information:**

The online version contains supplementary material available at 10.1007/s00442-021-04992-x.

## Introduction

The maintenance and enhancement of soil carbon are management imperatives for global sustainability (Minasny et al. 2017; Vermeulen et al. 2019). Grasslands are overachievers at storing soil carbon (Conant et al. 2017). For example, soil C storage is higher in temperate grasslands than temperate forests (Lal 2004) due to relatively high grassland plant biomass allocation to roots and relatively slow below-ground decomposition of these substrates (Jackson et al. 2017). Further, recent findings indicate that C storage is enhanced by increased grassland plant species richness in several situations: in systems recovering from disturbance (Yang et al. 2019), in experimental grasslands (Fornara and Tilman 2008, Steinbeis et al. 2008, Cong et al. 2014, Zou et al. 2019), and in natural ecosystems (Chen et al. 2018). These studies also find that plant life form and variation in species productivity influence C storage. Thus, management decisions to preserve grassland diversity have the potential to provide ecosystem services beyond the more obvious plant and wildlife conservation values.

A relationship between soil C and plant species richness is expected from niche theory. The co-occurrence of plant species with different niches (e.g., grasses vs forbs, native vs exotic, annual vs perennial) should lead to a more complete use of the available soil resources and overall higher carbon sequestration (i.e., niche complementarity; Turnbull et al. 2016). Higher soil C may also be related to the higher temporal stability often associated with richer plant communities: their many redundant species (i.e., the insurance effect), high likelihood that some species will do well when others do not (i.e., compensatory dynamics), and increased chance of selecting species that increase soil C from the “species pool” (i.e., the sampling effect) (Tilman 1999; Ives and Carpenter 2007) should help ensure that species-rich plant communities store carbon in all conditions and years. Underlying this expectation is the assumption that a diverse species mix will enhance the diversity and abundance of litter substrates for decomposition, and root carbon inputs that increase soil microbial activity and biomass (Lange et al. 2015). Moreover, positive feedbacks can occur between ecosystem functioning and species richness, where each new species may add to soil C and therein water holding capacity, in turn favoring the establishment of even more species (Chen et al. 2018; Hoffland et al. 2020; Werner et al. 2020). Importantly, a mechanistic link between soil C and species richness implies that increases or decreases in ecosystem services follow species gains or losses. Of course, variation in soil C across a landscape is not predicted by species richness alone; a more complete assessment should account for landscape-scale heterogeneity in factors like resource availability and disturbance history (Schimel et al. 1994).

Recent studies underline the importance of accounting for environmental heterogeneity for landscape-scale, observational studies, where control of environmental and disturbance factors is not feasible (Manning et al. 2019). For example, a recent meta-analysis of small-plot manipulations of species richness (i.e., plots ≤ 400 m^2^) shows that soil carbon storage generally increases with species richness (Weisser et al. 2017), while a meta-analysis of 35 observational studies on larger areas reveals about equal numbers of increased, decreased, and neutral relationships between soil C and species richness (van der Plas 2019). Here, we present a landscape-scale study of the soil C-species richness relationship and evaluate how the soil C-richness relates to landscape heterogeneity in grassland type, soil texture, and prairie dogs (*Cynomys ludovicianus*).

The three grassland types studied here (mixed grass prairies, xeric tallgrass prairies, and mesic tallgrass prairies) were adopted for management purposes long before our study began based on dominant plant species composition; so, an interesting question is whether these same designations correspond with differences in resource availability. These grassland types have the potential to vary substantially in topography, soil moisture, the composition of parent materials, and soil texture, which all affect soil C storage (Branson et al. 1965; Schimel et al. 1985; Hopkins-Arnold 1998). Soil texture, for example, influences carbon storage directly through variation in the degree of chemical and physical protection of soil organic matter, most often associated with clay content and indirectly through effects on soil available water that frequently controls plant productivity (e.g., Baldock and Skjemstad 2000; Hook and Burk 2000). Mixed grass prairie may have the lowest water availability due to surface clays and low soil organic matter (as described in our results), followed by xeric tallgrass (which, despite its name, is relatively wet deeper in the soil profile or where gravel mulch reduces evaporation), and with mesic tallgrass having the greatest water availability. The range of conditions of the different grassland types are further described in Table 1.

**Table 1:** Attributes of three grassland communities found on City of Boulder lands

Grassland type	Area (ha)	Most frequent native graminoid species	Land Use	Landscape context and composition	Moisture availability
Mixed grass prairie	4664	Western wheat (*Pascopyrum smithii* [Rydb.] Barkworth and D.R. Dewey) Blue grama (*Bouteloua gracilis* [Kunth] Lag. ex Griffiths) Sideoats grama (*Bouteloua curtipendula* [Michx.] Torr.) Buffalo grass (*Buchloe dactyloides* [Nutt.] Engelm.)	Livestock grazing Historic tilling Prairie dog occupied	Mosaic of diverse plant associations dominated by short- and mid-height species like western wheatgrass or needle and thread grass	Relatively dry, in part due to high clay content and low organic matter
Xeric tallgrass prairie	2310	Big Bluestem Sun sedge (*Carex pensylvanica* Lam.) Sideoats grama Blue grama	Livestock grazing	A tallgrass community that occurs in uplands on rocky soils, which can often overlay clay-rich subsoils. Characterized by tall grass species like big bluestem, little bluestem, and prairie dropseed, often intermixed with species characteristic of the Rocky Mountain montane life zone	Water stored at depth, available to deeply rooted natives
Mesic tallgrass prairie	140	Switchgrass (*Panicum virgatum* L.) Arctic Rush (*Juncus arcticus* Willd.) Big Bluestem Yellow Indian grass (*Sorghastrum nutans* [L.] Nash)	Irrigation Haying	A tallgrass community that occurs in floodplains and higher terraces where high ground-water tables or flood irrigation support big bluestem, switchgrass, and arctic rush	Relatively wet, with water available even at shallow depths

The presence of prairie dogs also varies spatially, affecting soil C storage directly through effects on soil structure and processes (Martinez-Estévez et al. 2013) and/or indirectly through changes in plant biomass, diversity, and composition (Beals et al. 2014). In our region, preliminary observations of prairie dogs suggest that their activities deplete native vegetation and lead to soil loss when colonies of these animals are constrained within a matrix of extensive agricultural and built environments (Seastedt 2013), while in other regions prairie dogs have been reported to increase soil C (Martinez-Estévez et al. 2013).

While research demonstrates that high species richness can enhance ecosystem functioning (Isbell et al. 2011; Tilman et al. 2012; Yang et al. 2019), managing for functional groups or single indicator species may be easier and more effective than managing for total species richness. For example, a key functional plant trait is the C_4_ photosynthetic pathway, most often associated with grasses. C_4_ grasses are expected to contribute more soil C than C_3_ grasses due to their relatively greater above-ground biomass and below-ground productivity, more recalcitrant tissues and slower decomposition, and higher nitrogen-use efficiency (Yang et al. 2019). However, results are conflicting, with some studies showing the presence of C_4_ grasses is associated with higher accumulated soil C (Fornara and Tilman 2008; O’Brien et al. 2010), while others show negative or no effect of C_4_ plant abundance on soil C pools (Mahaney et al. 2008; Hernández et al. 2013; Ampleman et al. 2014). In experimental manipulations, Fornara and Tilman (2008) demonstrated that higher species diversity plots were associated with greater soil carbon than lower diversity plots, even when C_4_ grasses were present in the lower diversity plots, suggesting the species richness and/or complementarity was a more important driver of carbon accumulation than individual functional groups. Another possibility is that an individual species may have a suite of traits that consistently favor soil C accumulation. If such species can also persist over long-time scales, its presence could hedge against losses of diversity that might reduce soil C. If the soil C-richness relationship differs among plant functional groups or when individual species are present, then managing for species richness within functional groups or managing for individual species will better serve the purpose of promoting carbon sequestration and managing for total species richness (c.f., Isbell et al. 2011).

Our study utilizes an extensive vegetation inventory across three grassland types comprising ~ 9700 ha of publicly managed lands to test two hypotheses:

### Hypothesis 1

Soil C is positively related to plant species richness, even when accounting for landscape heterogeneity in grassland type, soil texture, and prairie dogs.

### Hypothesis 2

Soil C shows even stronger relationships to richness or presence of plant species with adaptations expected to promote accumulation of soil C (e.g., native perennial grasses; species with C_4_ photosynthetic pathway).

## Materials and methods

### Study area

Grasslands managed by the City of Boulder, CO (USA) occupy the plains and foothill regions of Colorado’s Front Range centered at about 40 N latitude and 105 W longitude. The area experiences an average of 513 mm precipitation, with the spring-early summer interval being the wettest (NOAA 2020). Grassland inventory and monitoring efforts have documented about 800 vascular plant species (OSMP 2010). Unlike many other well-studied grasslands, the rainfall gradient generated by the adjacent Rocky Mountains along with substantial topographical differences and variation in parent material produce grassland communities with different dominant species across relatively short distances. These grasslands, therefore, host a unique and unusually diverse list of species (635 vascular species recorded in 9 years of monitoring), allowing for communities dominated by tallgrass species common to the Eastern US to lie adjacent to communities dominated by species of the shortgrass steppe or higher elevation montane plant communities (Vestal 1914; Livingston 1952; Branson et al. 1965; Moir 1969; Bock and Bock 1998). If these vegetation differences contribute to large local differences in carbon storage, Boulder grasslands would be a logical area to test the relationship of soil C and species richness.

The City of Boulder’s Open Space and Mountain Parks (OSMP) department’s Grassland Ecosystem Management Plan (OSMP 2010) describes many goals related to the accommodation of conservation, recreation, and historical agricultural uses. Plan implementation included mapping of vegetation to delineate plant alliances (USNVC Database Ver 2.02), aggregating alliances into grassland types (referred to as “conservation targets” in OSMP 2010), and the establishment of an ambitious monitoring program with emphasis on monitoring vegetation composition on the three upland grassland types of conservation interest not specifically targeted for agricultural use (Table 1). Some of these grassland types contain prairie dog colonies of varying densities (Johnson and Collinge 2004), which remain valued by some stakeholders for their role of keystone species (c.f. Kotlier et al. 1999).

### Vegetation monitoring

Vegetation transects were located using a Generalized Random-Tessellation Stratified Design (Stevens and Olsen 2004; R software) to achieve a random and spatially balanced design of 160 transects stratified across City of Boulder’s three grassland types. Between July and August of every sampling year, each transect was monitored using a point-intercept technique that recorded the top plant species or substrate intersected at 0.5 m on either side of every meter mark along a 50-m tape, giving a total of 100 possible intercepts. Intercepts were observed through an optical point projection device (Cover-Point, ESCO Associates) that magnified the point under the cross hairs of a lens, reducing bias in sampling (Buckner 1985). Cover for each species was estimated as the number of respective intercepts across the transect. Transect-level species richness was based on augmenting the species list created during point-intercept sampling with any additional species found while searching the entire 2 m × 50 m belt transect.

The magnitude of this effort (16,000 recordings per full data set) meant that not all transects could be sampled on an annual basis. Here, we use vegetation monitoring data from 2016, the last year in which nearly full vegetation sampling was available (158 of 160 total transects) prior to the initiation of soil sampling in 2018–2019. A complete vegetation data set collected in the prior year (2015) showed similar patterns when related to our soils data, even though 2015 was much wetter than 2016 (data not shown) and we, therefore, chose the most recent data set for our analyses.

### Soil sampling

Beginning in May of 2018, established vegetation transects were sampled for soils using a procedure that required accommodation for variations in rock cover and rock content. At each transect, a single, composited sample was obtained by sampling eight sites, four each at 10-m intervals on either side of each transect. Sampling locations were located approximately 2 m outside of the center vegetation transect line at each interval. We obtained a 2 cm diameter by 15 cm deep core at each site, producing a composited volume of about 375 cc of soil per transect. In the event that rocks precluded the use of a coring tool, a rock hammer was used to excavate a small pit, and approximately 50–60 cc of soil were scraped evenly across a 15 cm depth to produce a similar soil volume per sample. While we excluded rocks from our sampling, we acknowledge that plots with many rocks will simply store less Carbon per unit area. Records were kept of the number of probes attempted in obtaining each core (max = 15, at which time the rock hammer was used), and these data were subsequently recorded to be used as an index of site rockiness.

Samples were obtained and analyzed from 90 transects in 2018 and 59 additional transects in 2019 (149 total). Eleven of the 160 transects were not sampled and/or analyzed due to restrictions related to conservation, cultural resource protection, or major site disturbance. While sampling the top 15 cm tends to emphasize carbon deposition from grasses as opposed to shrubs or forbs (O’Keefe et al. 2019), our assumption here is that this sample represents an index of C found at these sites, an assumption that appears reasonable for grasslands in our area (e.g., Schimel et al. 1985). That study also showed that bulk density across prairie landscapes was relatively constant, implying that soil C values can represent an index of total C in the top 15 cm of soil.

Soils were air dried, root fragments and rocks removed by hand and passed through a 2-mm mesh sieve and stored until analysis. Soil texture (% sand, % silt, % clay) was determined using the Bouyoucos hydrometer method for analyzing the particle size of soils (Texas A and M 2005). A subsample of each soil was pulverized using a Cianflone model 2601 soil pulverizer (Scientific Instruments Corp.). These processed soils were then sent to the Soil, Water & Plant Testing Lab at Colorado State University where they were analyzed for inorganic C content using a pressure transducer (Sherrod et al. 2002) and analyzed for total C and N using the dry combustion method in a Leco furnace CHN Analyzer (Model LECO-CHN-1000). Organic C was calculated by subtracting inorganic C from total C in each sample; hereafter, when we refer to “soil C”, we are referring to % organic soil C.

### Prairie dog activity

Prairie dog activity around each transect was determined by ArcGIS spatial analyses as the spatial intersection of vegetation monitoring transects against the prairie dog colonies mapped in the field cumulatively between 1996 and the fall prior to soil sampling. To map prairie dog colonies, an annual field visit is conducted to mark the perimeter of the colony with a GPS and to confirm the presence of prairie dogs. Prairie dogs were considered present if any part of the transect fell within the boundaries of an active or historic prairie dog colony.

### Statistical procedures

#### Outliers

The final data used for analyses included 141 transects sampled and analyzed for both soils (2018–2019) and vegetation (2016) that remained after excluding outliers that appeared contaminated with unusual amounts of C, N or both materials, although we cannot be sure. The 141 samples reported were those found within three standard deviations of the corresponding mean (i.e., for C, N, or C:N ratio) calculated without inclusion of obvious outliers from the pool of samples. We used SAS (Statistical Analysis System, SAS 9.4, 2017) and R (3.6.0) as our primary analysis tools.

#### Linear models

For simplicity, we used soil C as our response variable whenever possible, although we acknowledge that the mechanistic relationship between soil C and plants is a continuous feedback and therefore edaphic or botanic variables could be used as the response variable. We prefer to use soil C as the response variable because we want to isolate conditions/predictors that managers can change to protect or influence carbon sequestration. Further, we note that soil N is correlated with soil C (*r*^2^ = 0.87), and the ratio of soil C to N would be of interest to some readers, but we leave this unexplored. Finally, we acknowledge that soil C is involved in complex multi-way feedbacks with environmental traits (e.g., climate, topography, land use) and ecosystem functions (e.g., productivity, herbivory, biodiversity) (e.g., Weisser et al. 2017; van der Plas 2019) beyond the set of predictors we have chosen to measure here.

**Fig. 1:**
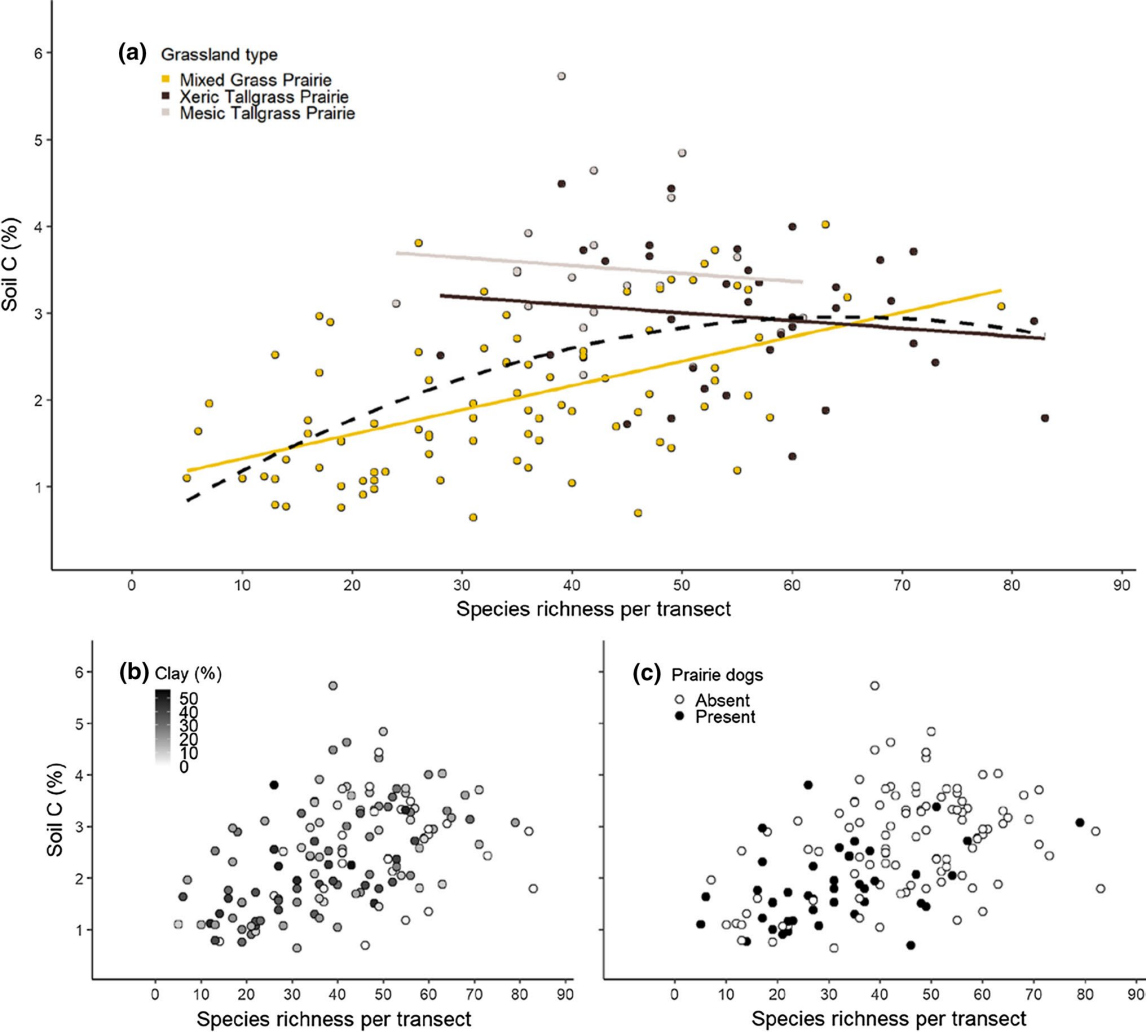
Relationship of soil C and plant species richness, colored by each of three predictors: **a** grassland type, **b** soil texture, and **c** prairie dog presence. In panel **a**, the solid lines represent the slope parameters from linear regression; the dashed line represents the polynomial fit

For Hypothesis 1, we derived four predictors (species richness, grassland type, clay, and the presence/absence of prairie dogs) for each transect. To test H1, we fit 6 models (Table 2), including a “full model” with all four predictors, 4 constituent models treating each single variable as a predictor, and the best multivariate model. To find the best multivariate model, we ran a candidate set of 33 models and selected the one with the lowest AIC score (see Electronic Supplementary Material 1 for list of models and AIC values). There appeared to be a non-linear relationship between soil C and species richness, and so we fit a model with a polynomial term for species richness (Fig. 1). Finally, we fit 6 additional models to describe relationships among our four predictors themselves.

**Table 2:** Statistical models explaining variance in soil C measured among 141 grassland transects

Hypothesis	Model #	Model description	Model formula with *P* values^a^	*R*^2^	AIC	df model, df error
1	1	Full model	Soil C–species richness^***^ + grassland type^***^ + clay^ns^ + prairie dog presence/absence^ns^	0.41	337.3	5, 135
1	2	Constituent model 1	Soil C–species richness^***^	0.21	374.3	1, 139
1	3	Constituent model 2	Soil C–grassland type^***^	0.33	351.7	2, 138
1	4	Constituent model 3	Soil C–clay^*^	0.03	403.8	1, 139
1	5	Constituent model 4	Soil C–prairie dog presence/absence^***^	0.14	386.9	1, 139
1	6	**Best model**	Soil C–species richness^***^ + grassland type^***^ + species richness X grassland type^**^	0.44	330.0	5, 135
2	7	Models for species subsets	Soil C–native species richness^***^	0.17	381.2	1, 139
2	8	“	Soil C–exotic species richness^**^	0.04	402.1	“
2	9	“	Soil C–native perennial graminoid species richness^***^	0.28	361.5	“
2	10	“	Soil C–exotic perennial graminoid species richness^***^	0.09	394.9	“
2	11	“	Soil C–native perennial forb species richness^***^	0.11	391.4	“
2	12	“	Soil C–exotic perennial forb species richness^**^	0.06	398.9	“
2	13	“	Soil C–native annual forb species richness^ns^	0.0	408.7	“
2	14	“	Soil C–exotic annual forb species richness^ns^	0.0	409.2	“
2	15	“	Soil C–*Andropogon gerardii* 1/0^***^	0.27	364.6	“

**Fig. 2:**
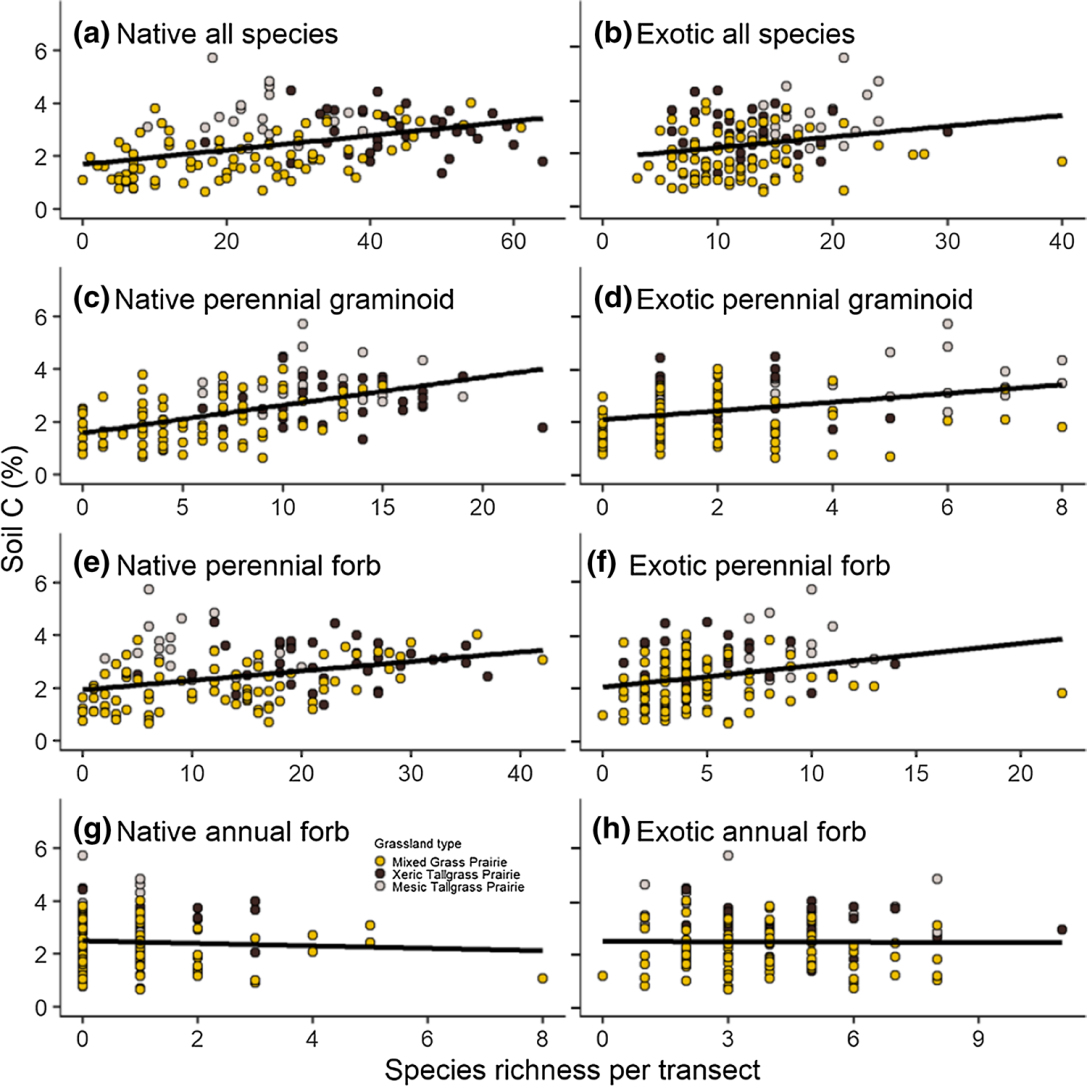
Relationship between soil carbon and plant species richness in three different grassland types for natives (left column) and exotics (right column) by group (rows). Data are presented for: **a**, **b** all species, **c**, **d** perennial graminoid, **e**, **f** perennial forb and **g**, **h** annuals. See Table 2 for statistics. Note that the x axis scales differ for each plot

See Figs. 1 and 2 for graphical depictions of relationships and more statistical information. See ESM2 for the data file for use in re-running these models to investigate the coefficients and effect sizes

The best model is in bold

^a***^P < 0.001; ^**^P < 0.01, ^*^P < 0.05, *ns* not significant

Clay was negatively correlated with species richness (*r* = − 0.33). Thus, we used variance partitioning to apportion the variation in soil C among these two predictors (species richness and clay) as well as the joint/shared effects of the two predictors (Peres-Neto et al. 2006). We used the “varpart” function of the “vegan” package in R to fit three linear models (soil C–species richness; soil C–clay; soil C–species richness + clay) for this purpose. The procedure then separates the fractions using addition and subtraction as applied to the model adjusted R^2^ values.

For Hypothesis 2, we derived 9 predictor variables: native species richness, exotic species richness, species richness for 6 functional groups (native perennial graminoid [grasses, sedges, rushes], exotic perennial graminoid, native annual forb, exotic annual forb, native perennial forb, exotic perennial forb), and the presence/absence of big bluestem (*Andropogon gerardii* Vitman) Note: native and exotic annual graminoids were excluded due to their rarity in the dataset; likewise, species classified as “other” (cacti and woody species) were excluded. We chose to focus on *A. gerardii* (versus other indicator species) for several reasons: (1) it has likely effects on soil C, based on literature showing that C_4_ grasses contribute disproportionately to soil C (O’Brien et al 2010; Fornara and Tilman 2009), (2) its cover is considered an indicator of the condition of our grasslands in our grassland management plan (OSMP 2010), and (3) it is frequent and abundant in our grasslands. Cursory analysis using linear regression (not shown) showed that *A. gerardii* had the strongest relationship to soil C of all species in our dataset (almost 2 × stronger that the 2nd best predictor species).

In summary, to test Hypothesis 2, we fit 9 models (Table 2): two models predicting soil C from species richness of native or exotic species, 6 models predicting soil C from richness of the 6 functional groups, and one model using the presence/absence of *A. gerardii*.

The models used to test our main hypotheses were fit using linear models and least sum of squares (“lm” function in R). We tested if each model met the assumptions of normality of residuals and homogeneity of variance. The assumptions were met for just 6 of the 15 models, but model assumptions could be met via variable transformations or non-parametric tests for all the remaining 9 models, and these adjustments made no difference to the assessment of variable significance (not shown). Therefore, for convenience, we share the results from using conventional linear models and untransformed variables.

The derived dataset is available in csv format (ESM2).

## Results

### Hypothesis 1

Soil C is positively related to plant species richness, even when accounting for landscape heterogeneity in grassland type, soil texture, and prairie dogs.

### Soil C–species richness

We found that soil C was related to species richness (*R*^2^ = 0.21, *P* < 0.0001; Fig. 1). A curvilinear polynomial fit, implying that soil C was highest at intermediate richness, accounted for 4% more of the variance than did the linear response (*R*^2^ = 0.25; Fig. 1a) and had a lower AIC value (368.4 vs 374.3).

### Soil C–grassland type

Soil C was significantly related to grassland type (*R*^2^ = 0.33; Table 2), where mesic tallgrass prairies had the highest soil C (Table 3). Adding grassland type to the model of species richness more than doubled the regression *R*^2^ values (Fig. 1), and a model that included an interaction of species richness and grassland type (ESM1) explained 43% of the variation in soil C. Three candidate models were tied (i.e., within ± 2 AIC units of each other) for “best model”, but based on parsimony, we selected the model with the fewest parameters as our best model: soil C ~ species richness * grassland type. The two other competing models had the additional predictors of prairie dog presence/absence and clay (ESM1) suggesting that prairie dogs and soil clay have a role to play even after accounting for species richness and grassland type.

**Table 3:** Average soil and plant characteristics of three grassland communities

Grassland Type	Soil clay (%)	Rockiness (# of core attempts)	Soil C (%)	Species richness
Mixed grass prairie	24.8	3.0	2.0	33.8
Xeric tallgrass prairie	9.6	10.9	3.0	56.0
Mesic tallgrass prairie	12.8	6.4	3.5	43.6

Of note is that when the relationship between soil C and plant richness is analyzed separately by grassland type, only the mixed grass prairie exhibited a significant positive relationship (*R*^2^ = 0.26, *P* < 0.001). The other two grasslands exhibited no pattern between soil C and richness (Xeric: *P* = 0.41; Mesic *P* = 0.69). It is possible that the lower sample size and limited range in species richness across xeric and mesic tallgrass prairies prevented the detection of a soil C–richness relationship.

### Soil C–soil texture

Soil texture, as measured by % clay, was significantly but weakly negatively related to soil C (*R*^*2*^ = 0.03; Table 2). Variance partitioning showed that clay had a much smaller effect on soil C than did species richness: unique effect of clay on soil C, *R*^2^ = 0.0; unique effect of species richness on soil C, *R*^2^ = 0.18; shared effect of clay and species richness on soil C, *R*^2^ = 0.04. These results suggest that texture was not driving patterns of soil C.

### Soil C–prairie dogs

The past or current presence of prairie dogs produced a significant decline in soil C from 2.7% to 1.8% (*R*^2^ = 0.14; *P* < 0.001; Fig. 1). Prairie dogs were not included in the best model, likely due to the non-orthogonal nature (i.e., correlation) between the predictors, as described below.

### Correlation among species richness, grassland type, soil texture, and prairie dogs

Six additional statistical models describe the relationship among our four predictors:

- Species richness was significantly related to grassland type (*R*^2^ = 0.31, *P* < 0.001)
- Species richness was negatively related to clay (*b* = − 0.40; *R*^2^ = 0.10; *P* < 0.001)
- Species richness was negatively related to prairie dog presence (*b* = − 13.5; *R*^2^ 0.13; *P* < 0.001)
- Grassland type was significantly related to clay (*R*^2^ = 0.24, *P* < 0.001)
- Grassland type was significantly related to prairie dogs (they were found almost exclusively in mixed grass prairies; chi-squared test: *X*^2^ = 29.5; *P* < 0.001).
- Clay was positively related to prairie dog presence (*b* = 7.2, *R*^*2*^ = 0.05; *P* < 0.01)

#### Hypothesis 2

Soil C shows even stronger relationships to plant species richness or presence of species with adaptations expected to promote accumulation of soil C.

**Fig. 3:**
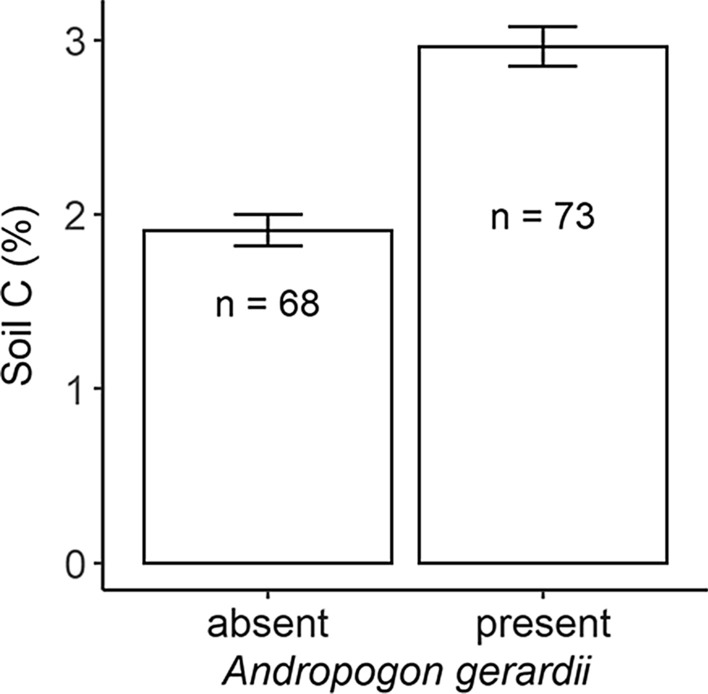
Soil carbon (mean ± SE) by the presence of *Andropogon gerardii* (big bluestem) (mean ± standard error). *A. gerardii* was present in 73 of the 141 transects

The relationship between soil C and species richness for various combinations of functional groups often resulted in positive relationships with soil C (Fig. 2). The strongest contribution to a soil C relationship was created by native perennial graminoids (*R*^2^ = 0.28), but the surprising finding was that the presence or absence of a single C_4_ species, *A. gerardii*, was an equally strong predictor (*R*^2^ = 0.27; note, species richness was also correlated with the presence/absence of *A. gerardii*; *R*^2^ = 0.41). All other groupings either contributed less variance to the relationship or were non-significant (Fig. 3).

Both exotic and native species richness show the overall significant positive relationship with soil C, however the strength of the relationship is much weaker with exotic species (*R*^2^ = 0.04) versus natives (*R*^2^ = 0.17). Of interest, there was no correlation between native and introduced species richness (*P* = 0.64).

## Discussion

We found a positive relationship between soil C and plant species richness, despite confounding effects of landscape heterogeneity. The high soil C storage in our species-rich communities may be due to a high degree of niche complementarity of functional groups (Fornara and Tilman 2008; Turnbull et al. 2016; Yang et al. 2019), relatively long temporal stability of plant communities (Hector et al. 2010), positive feedbacks of species richness on carbon accumulation as mediated by increased productivity, high above-ground plant and root biomass (Yang et al. 2019), increased diversity of soil organic compounds (El Moujahid et al. 2017) enhanced microbial activity and diversity (Lange et al. 2015), or some other explanation. Regardless of the exact mechanistic cause of the relationship, the link that we discovered between soil C and species richness implies that addition or loss of ~ 35 species per 100 m^2^ is associated with the addition or loss of 1% soil C.

The relationship between soil C and plant richness was much better described by accounting for grassland type, soil texture, and prairie dogs. Within grassland types, only one community type (mixed grass prairie) showed a strong relationship between soil C and species richness, but it has twofold higher acreage than the next most widespread grassland type (Table 1). Transects in mixed grass prairie spanned a relatively broad range of soil C and species richness, including the lowest observed values of C and richness associated with land use legacy effects such as tilling and overgrazing, which may have leveraged the overall soil C–richness relationship. If one interprets C storage as the integrated outcome of inputs and outputs, our results argue that the mixed grass prairie provides fewer inputs due to a combination of factors including reduced species richness, a larger cover by introduced species (which lowers native diversity and contributes relatively small amounts of organic matter), reduction in productivity due to land use legacy effects and direct and indirect effects of prairie dogs. These potential factors are consistent in both explaining the mixed grass prairie’s reduced soil C content relative to the other two communities, and the pattern observed between soil C and richness within the community itself. According to results from biodiversity experiments, these low diversity communities may be expected to have relatively larger losses in productivity (and corresponding declines in soil C) when species are lost as compared to more species-rich communities (Cardinale et al. 2011). In contrast, we suspect that the tallgrass communities are potentially more resource rich, have higher plant available water (Branson et al. 1965), and have been less disturbed over time by historical cattle grazing, tilling and long-term prairie dog occupation.

Some parallels exist between our findings and those of Konza Prairie, a tallgrass prairie site where a landscape gradient generates higher productivity and higher amounts of carbon storage in relatively species-poor mesic lowlands when compared to species-rich xeric tallgrass uplands (Gibson and Hulbert 1987; Briggs and Knapp 1995; Collins and Calabrese 2012). At that tallgrass site, plant competition for light in productive areas likely restricts a subset of species, and this interpretation may explain the differences observed between the xeric and mesic tallgrass prairies in our study, where plant productivity levels in mesic tallgrass can match or exceed those at the Kansas lowland site (Hopkins-Arnold 1998). Xeric tallgrass sites have the highest richness, but mesic tallgrass sites have the highest soil C. Both the tallgrass prairies have higher richness and soil C than the mixed grass prairies, regardless of the presence or absence of prairie dog communities. The result that species richness peaks at intermediate levels of soil C is reminiscent of the “humped-back” model used to describe the richness-production relationship observed elsewhere (c.f., Adler et al. 2011; Fraser et al. 2015). The non-linear pattern also indicates that total richness is not the most relevant predictor of soil C in tallgrass prairies.

The inverse relationship between soil C and soil clays on these sites was a surprise, but it is worth restating that the importance of clay to soil C in our study was very small. At a regional scale, soil carbon storage in mesic regions is often positively related to soil clay content (e.g., Burke et al. 1989; Schimel et al. 1994; Jobbágy and Jackson 2000). This phenomenon assumes that soil C becomes physically protected from further decomposition by sorption to mineral surfaces and aggregate formation. However, there is a limit to the capacity for clays to protect organic C in some environments and not all clay particles are equal in their ability to stabilize soil C because of their diverse mineral properties (Hassink 1997; Percival et al. 2000; Rasmussen et al. 2018). Thus, while we expect that clay content plays a role to enhance carbon storage in our study, the effect is obscured by other factors. At reduced precipitation levels (i.e., below 34 cm of annual precipitation), high clay content in soils can have a negative effect on plant production because a relatively higher percentage of soil water is lost to surface evaporation (Sala et al. 1988). However, spring and summer growing season precipitation almost always exceeds 34 cm at our sites (https://psl.noaa.gov/boulder/Boulder.mm.precip.html). Given an average rainfall input of ~ 50 cm in this area, increased clay should support increased nutrient storage and availability that would result in greater plant species richness, leading to greater C deposition in the soil. This is not the case, and we speculate that the increased surface clays, along with the increased bare surfaces found in the mixed grass sites, result in greater surface evaporation and water runoff characteristic of shale-derived soils (Branson et al. 1965), and therefore reduced soil water storage. Less soil C can also reduce water storage (Werner et al. 2020) thereby further reducing plant richness. In any event, the sandier soils will move water deeper into the soils where C_4_ plants in particular might be able to access this resource, resulting in higher plant productivity and richness and subsequently greater C content of soils. Again, we note that the relationship between soil C and clay was only a weak negative correlation in our study, but it is at least fair to say that carbon storage was not positively related to soil clay content.

Prairie dogs are a keystone species (Kotliar et al. 1999), and an important component of high functioning native-dominated grasslands in our region. Some colonies support intact native plant communities and prairie dog presence provides prey and landscape structure necessary for the presence of associated species (OSMP 2010). However, in our study, prairie dog occupied sites had significantly lower soil C, although we note the prairie dog effect on soil C is difficult to disentangle from the effects of species richness and soil clay. Some prairie dog colonies in our area are characterized by a high density of burrows and diminished native vegetation, likely related to low predator pressure and the restriction that urbanization places on prairie dog movement (OSMP 2010). These conditions have led to localized loss of topsoil from prairie dog colonies (Seastedt et al. 2013), leaving behind the C-depleted soils that we measured here. A different study on prairie dogs and soil C reported an increase soil C related to the burying of plant material, but further examination of the experimental design and sample analysis indicate that the elevated levels of soil C at depth was the result of a layer of calcium carbonate common in arid and semi-arid environments (Martinez-Estévez et al. 2013).

Our best model explained 43% of the variation in soil C, leaving much of the landscape variation of soil C unexplained. Land uses, such as fire, grazing and tilling history may account for variation in soil C in our grasslands, as can heterogeneity in soil chemical properties, parent material, landscape position, presence of other functional groups (e.g., legumes), and plant productivity (Conant et al. 2017; Jackson et al. 2017; Rasmussen et al. 2018).

Positive soil C–richness relationships were observed within various functional groups, though these were often weaker than relationships between soil C and total species richness, with the one exception of the C4 dominant, *A. gerardii*. The strong soil C–richness relationship for native perennial graminoids reflects the diverse niches of member species. When richness is high, the mix of grasses, rushes, and Cyperaceae species in this group can likely exhibit complementarity in both time (e.g., early-season and late-season species; varying rates of litter decomposition among C_3_ and C_4_ species) and space (e.g., microhabitat variation in soil moisture availability; separation by rooting depth), thus enhancing carbon accumulation, and reflecting the major importance of graminoids to soil C pools (February et al. 2020).

The similarly strong relationship between soil C and *A. gerardii* suggests that big bluestem, like other C_4_ grasses, contributes disproportionately to soil C (O’Brien et al 2010; Fornara and Tilman 2009). For *A. gerardii* in our study area*,* late-season physiological activity during hot, dry conditions that trigger dormancy in other plants, may extend the period of soil C accrual, while plasticity in rooting characteristics may facilitate root exploitation of microhabitats (Weaver and Darland 1949). High water use efficiency of this species can ensure high rates of carbon gain (Turner et al. 1995), even when these tallgrass communities experience seasonal water stress and periodic drought. These traits, along with tall stature and relatively high above-ground productivity, relatively slower turnover and high C:N ratios of shoot, fine root and coarse below-ground structures (Wedin and Tilman 1990; Craine et al. 2003) and significantly higher fine root biomass when compared to other tallgrass species (Craine et al. 2003) suggest that *A. gerardii* contributes high C:N carbon sources through both above- and below-ground parts and has a suite of functional traits favoring high C storage. This relationship was not merely an artifact of the strong correlation between *A. gerardii* presence and species diversity or its utilization of the C_4_ photosynthetic system. The positive effect of *A. gerardii* on soil C may best be observed in grassland sites with a long history of *A. gerardii* occupation such as ours, as compared to younger, restored agricultural sites where the contribution of C_4_ species to soil C lags behind C_3_ species more abundant early in succession (Mahaney et al. 2008; Hernández et al. 2013). These relationships provide general support that some plant groups and some species appear more important than others in the C storage process.

Soil C was much more strongly related to native species richness than to exotic species richness, and, in turn, native species richness was not related to exotic species richness. These relationships are potentially at odds with the literature (e.g., Stohlgren et al. 2003). Normally, what benefits native species richness benefits introduced species richness (Lonsdale 1999). In our case, we expected that exotic species richness would be favored in ruderal areas with disturbance, high surface clays, and poor soils, and competitively excluded from resource-rich, intact tallgrass prairies. Our finding means that exotic species make only minor contributions to soil C in these grasslands.

## Conclusion

Surveys such as ours argue that careful analysis of landscape variables can deepen our understanding of the relationship between ecosystem services and changing plant species richness.

Maintaining soil organic matter in a semi-arid environment by way of plant conservation management practices appears to increase C, nutrient storage and release and increase water holding capacity, but high surface clay content and disturbance by prairie dogs can provide a major challenge to maintaining both soil C and species richness. However, exotic species richness was lower and less variable than native species richness; as a result, we may have underestimated the contributions of exotic species to soil C.

## Supplementary Information

Below is the link to the electronic supplementary material.

Supplementary file1. Best model selection (DOCX 18 KB)

Supplementary file2. Data file in csv format (CSV 10 KB)

Supplementary file3. Metadata file is csv format (CSV 4 KB)

## Data Availability

The data are available as ESM2.

## Code availability

The code used during the current study is available from the corresponding author on reasonable request.
