# Supplementary material for: Soil carbon and plant richness relationships differ among grassland types, disturbance history and plant functional groups
Source: Oecologia. 2021 Jul 25;196(4):1153–66. doi: 10.1007/s00442-021-04992-x (PMC8367897; doi:10.1007/s00442-021-04992-x)
Supplement: Supplementary file 1 — Supplementary file1. Best model selection (DOCX 18 KB) [file 442_2021_4992_MOESM1_ESM.docx]

Electronic Supplemental Material (ESM) 1. Best model selection.

| **Model** | **AIC** |
| --- | --- |
| soilc ~ r * grasslandType + clay | 329.4870 |
| soilc ~ r * grasslandType | 330.1192 |
| soilc ~ r * grasslandType + pd | 330.2156 |
| soilc ~ r * clay + grasslandType | 335.9884 |
| soilc ~ r + grasslandType + clay + pd | 337.2617 |
| soilc ~ r + grasslandType + clay | 337.5171 |
| soilc ~ r + grasslandType + pd | 338.0141 |
| soilc ~ r + grasslandType | 338.2329 |
| soilc ~ r + grasslandType * pd | 339.7515 |
| soilc ~ r * pd + grasslandType | 340.0055 |
| soilc ~ r + grasslandType * clay | 341.3267 |
| soilc ~ grasslandType + pd | 349.1915 |
| soilc ~ grasslandType + clay * pd | 349.3783 |
| soilc ~ grasslandType + clay + pd | 349.5436 |
| soilc ~ grasslandType * pd | 350.8085 |
| soilc ~ grasslandType * pd + clay | 350.9617 |
| soilc ~ grasslandType | 351.6519 |
| soilc ~ grasslandType + clay | 352.1310 |
| soilc ~ grasslandType * clay + pd | 353.2556 |
| soilc ~ grasslandType * clay | 355.6901 |
| soilc ~ r + clay * pd | 364.8588 |
| soilc ~ r + pd | 365.9912 |
| soilc ~ r * clay + pd | 367.0598 |
| soilc ~ r * pd | 367.8828 |
| soilc ~ r + clay + pd | 367.9689 |
| soilc ~ r * pd + clay | 369.8732 |
| soilc ~ r | 374.3303 |
| soilc ~ r + clay | 376.0292 |
| soilc ~ r * clay | 376.0478 |
| soilc ~ clay * pd | 379.5974 |
| soilc ~ pd | 385.9716 |
| soilc ~ clay + pd | 386.1356 |
| soilc ~ clay | 403.8357 |
